# Evaluation of the role of serum soluble ST2 as a diagnostic biomarker for cancer-associated venous thromboembolism

**DOI:** 10.1016/j.htct.2025.103740

**Published:** 2025-02-18

**Authors:** Eman Mohamed Abdellatif, Emad Hamdy Hamouda Mohammed, Azza Mohamed Amin Darwish

**Affiliations:** Faculty of Medicine, Alexandria University, Alexandria, Egypt

**Keywords:** Venous thromboembolism, Cancer, ST2, D-dimer

## Abstract

**Introduction:**

Venous thromboembolism, a common complication associated with cancer, causes increased morbidity and mortality. D-dimer levels are often increased non-specifically in cancer which limits their use to diagnose venous thromboembolism. The current study aimed to investigate the role of the serum soluble suppression of tumorigenicity 2 (sST2) as a new biomarker to diagnose venous thromboembolism in cancer.

**Methods:**

Eighty-eight patients with different types of cancer were enrolled and divided into two groups: Group I: 44 cancer patients with confirmed diagnosis of venous thromboembolism and Group II: 44 age- and sex-matched cancer patients without any thrombotic complications. The D-dimer test and sST2 measurement were performed for all study subjects.

**Results:**

Serum sST2 levels were significantly higher in Group I than in Group II (*p*-value < 0.001); the median serum sST2 was 13.02 ng/mL (range: 7.65–117.9 ng/mL) in Group I versus 8.56 ng/mL (range: 5.59–10.33 ng/mL) in Group II. There was a significant positive correlation between serum sST2 and the D-dimer level. Using a receiver operating characteristic curve, sST2 had a greater area under the curve than the D-dimer test (0.974 versus 0.869, respectively). Although the D-dimer test was more sensitive, sST2 had a greater specificity than D-dimer (95.45 % versus 27.3 %, respectively) and a higher positive predictive value (95.3 % versus 56.8 %, respectively).

**Conclusion:**

The results of the current study support a potential role of soluble ST2 to aid in diagnosing venous thromboembolism in cancer patients.

## Introduction

Thrombosis has been established as a common complication occurring in cancer patients and a major cause of death in these patients.^1^ The reported incidence of cancer-associated thrombosis (CAT) varies widely between studies due to differences in the studied populations, cancer type, cancer stage, tumor-directed therapy, patient related risk factors and comorbidities. However, the incidence of CAT is increasing over time.^2^ The most common type of CAT is venous thromboembolism (VTE) which includes deep vein thrombosis (DVT) and pulmonary embolism (PE). Cancer-associated VTE is highly consequential for patients with cancer, associated with worse survival, increased morbidity, prolonged hospitalization and potential interruption of systemic cancer therapy.^3^ Cancer patients have multiple abnormalities in each component of Virchow's triad, contributing to various thrombotic complications. These abnormalities include: prolonged immobility and compression of blood vessels by tumor which lead to stasis of blood flow.^4^ The hypercoagulable state can be attributed to release of tumor cytokines, recent major surgery, effect of chemotherapy and the use of erythropoiesis-stimulating agents. Endothelial injury is mainly caused by direct invasion by the tumor, presence of central venous catheters, chemotherapy and radiotherapy.^4^^,^^5^

D-dimer is produced by the action of fibrinolytic enzyme plasmin on cross-linked fibrin. High levels of D-dimer indicate activation of both coagulation and fibrinolytic systems, and so this biomarker is used for the diagnosis of VTE. However, elevated D‐dimer can also be observed in acute or chronic inflammatory disorders, following recent surgery, major trauma, during pregnancy, liver diseases and various types of cancer regardless of VTE occurrence.^6^ In cancer patients, the D-dimer test has a high sensitivity but it lacks the specificity needed to confirm the diagnosis and so it is mainly useful for exclusion of VTE. Thus, most of cancer-associated VTE patients require imaging studies for diagnosis.^7^ Recent research efforts have focused on the utility of different plasma components with greater specificity than D-dimer as novel biomarkers to help confirming or excluding VTE diagnosis in cancer patients. This is especially important when imaging studies are difficult to perform because of disease limitations. These markers include: *p*-selectin, E-selectin, microparticles, thrombin, fibrinogen, interleukin-10 and other inflammatory markers.^8^

Suppression of tumorigenicity 2 (ST2), also known as interleukin-1 receptor-like-1 (IL1RL1), is one of the recently studied inflammatory markers. It is one of the toll-like receptors. ST2 with its two isoforms; (transmembrane [ST2 L] and soluble [sST2]) is mainly found in endothelial cells and cardiac muscle cells.^9^ ST2 L, activated by interleukin 33 (IL33), can, in turn, activate mitogen-activated protein kinase (MAPK) and nuclear factor kappa B (NF-kB) signaling, resulting in the activation and regulation of cellular immune response, and the inflammatory cascade. The truncated soluble form of ST2 (sST2), which will be investigated in this study, prevents the signaling of ST2 L by competitive binding with IL33.^9^^,^^10^ A new connection has been shown between inflammation and the coagulation system through the stimulatory effect of IL33 on tissue factor, the primary trigger activating the coagulation cascade.^11^

Studies found that the level of sST2 is significantly elevated in many disorders such as pneumonia, bronchial asthma and graft-versus-host disease.^10^^,^^12^^,^^13^ Elevation of sST2 has been recognized as a poor prognostic marker in myocardial infarction.^9^^,^^14^ In atrial fibrillation patients, sST2 was found to be strongly associated with subsequent stroke and systemic embolization.^15^ The present study was designed to evaluate the role of sST2 as a new biomarker in the confirmation or exclusion of VTE in cancer patients, and to study the association between this marker and D-dimer as a marker of hypercoagulability in these patients.

## Methods

### Study subjects and study design

Eighty-eight cancer patients of different types were enrolled in this case-control observational study. They were recruited from the oncology clinic and the intensive care unit of Alexandria Main University Hospital. The recruited patients were divided into two groups: Group I (VTE group) which included 44 cancer patients with confirmed diagnosis of VTE (first episode of DVT or pulmonary embolism), and Group II (non-VTE group) which included 44 age- and sex-matched cancer patients without any thrombotic complications. Patients with history of previous thrombosis, patients with duration of symptoms more than ten days, pregnant women, patients with history of infection or surgery in the previous four weeks and those with ongoing anticoagulation were excluded from the study. Patients were recruited consecutively, according to these inclusion and exclusion criteria, from December 2023 to April 2024. The present study was reviewed and approved by the Ethics Committee of the Faculty of Medicine, Alexandria University (IRB 00012098, FWA 00018699, serial number 0,306413). Written informed consent was obtained from every subject before entering the study.

All patients underwent full history taking, weight and height measurement with body mass index calculation and a precise clinical examination. Information was obtained from all patients regarding the presence of other thrombotic risk factors (e.g. prolonged immobilization, recent surgery, hormone replacement therapy or the use of contraceptive pills, presence of central venous catheter). Data of relevant radiological and histopathological examinations were also collected from all patients. The diagnosis of cancer depended on a core biopsy (or post-surgical excisional biopsy) and histopathological examination with an assessment of the histopathological type, grade and lymphovascular invasion. Diagnosis of DVT was confirmed by duplex ultrasonography and diagnosis of PE was confirmed by a contrast-enhanced spiral computed tomography (CT) scan or CT angiography.

All subjects enrolled in Group II had no signs or symptoms of any thrombotic complications. Using the Khorana score for estimating VTE risk,^16^ 77.3 % of non-VTE patients were low-risk patients and 22.7 % were at intermediate risk. Raw data, including all the patients’ medical and laboratory data, are available upon request.

### Laboratory assays

Routine laboratory investigations were performed for all patients with peripheral venous blood being collected into three tubes: an EDTA (ethylene diamine tetra acetic acid) tube for complete blood count (CBC) testing, a sodium citrate tube for D-dimer testing and a plain (non-anticoagulated) tube for the remaining tests which included liver function tests (aspartate aminotransferase [AST), alanine aminotransferase [ALT]), renal function tests (serum urea and creatinine) and soluble ST2 measurement. The CBC was performed on an ADVIA2120i hematology analyzer (Siemens Laboratory Diagnostics, Germany). Liver function and renal function tests were performed on an ADVIA 1800 chemistry analyzer (Siemens Laboratory Diagnostics, Germany) and D-dimer testing was performed on a CS2100i automated blood coagulation analyzer (Sysmex corporation, Japan). Serum sST2 was measured using a human sST2 enzyme-linked immuno-sorbent assay (ELISA) kit (Bioassay Technology Laboratory, China - Cat number E4287Hu).

Serum sST2 measurement: Serum was isolated by centrifugation for 20 min at 3000 RPM and then stored at −80 °C until use. The assay principle is a sandwich immunoassay technique where the ELISA plate has been pre-coated with human sST2 antibodies to bind with the serum sST2 present in the sample. Five standard solutions were prepared by serially diluting the provided standard stock solution with a standard diluent. The readings of standard solutions were used to construct a standard curve by plotting the optical density (OD) for each standard against its concentration and drawing a best fit curve using a computer-based curve-fitting software. The test was performed according to manufacturer instructions. In brief, serum was added, a wash was performed, then biotinylated human sST2 antibody reagent was added to bind previously captured serum sST2. Then, streptavidin-horseradish peroxidase was added. After incubation, unbound horseradish peroxidase was washed away, a substrate solution was added and color developed in proportion to the amount of serum sST2 present. The reaction was stopped by adding stop solution, then absorbance was measured at 450 nm using a Stat Fax 2100 Microplate Reader (Awareness Technology, USA). According to the manufacturer, the intra-assay and inter-assay coefficients of variation of the test are <8 % and <10 %, respectively.

### Statistical analysis

Data were fed into a computer and analyzed using the IBM Statistical Package for Social Sciences (SPSS) software package version 20.0. (Armonk, NY: IBM Corp). The chi-square test was applied to compare qualitative data of the two groups. Alternatively, Fisher's exact test or the Monte Carlo correction test was applied when >20 % of the cells had an expected count of <5. Quantitative data were tested for normality using the Shapiro-Wilk and Kolmogorov-Smirnov tests. If normally distributed, Student *t*-test was used to compare the two groups. If not normally distributed, the Mann-Whitney test was used, while the Kruskal-Wallis test was used to compare more than two groups. Regarding correlation studies, the Spearman coefficient was used to correlate between non-normally distributed quantitative variables. The Mann-Whitney and Kruskal-Wallis tests were used to correlate D-dimer and sST2 (non-normally distributed quantitative data) with different qualitative parameters. A receiver operating characteristic (ROC) curve was constructed to determine the diagnostic performance of the studied markers. Significance of the obtained results was considered at a 5 % level (*p*-value ≤0.05).

## Results

Of the 88 participating patients, 47 were female and 41 were male. Their age ranged from 38 to 74 years and the mean BMI was 25.20 ± 5.90 kg/m^2^. Clinical history of the patients revealed that 35.2 % of the participants had hypertension, 37.5 % had diabetes mellitus, 9.1 % had varicose veins, 37.5 % were smokers, 55.7 % had undergone surgical tumor excision and 43.2 % were on systemic chemotherapy regimens. The recruited cases included colon cancer (25 cases), breast cancer (29 cases), gastric cancer (14 cases), bladder cancer (6 cases), lung cancer (6 cases), prostate cancer (3 cases) pancreatic cancer (3 cases), ovarian cancer (1 case) and brain cancer (1 case). A total of 51.1 % of the cases were in cancer Stage I-II and 48.9 % were in Stage III-IV. Study subjects were divided into Group I (44 patients aged 44–74 years; 20 males and 24 females) and Group II (44 patients aged 38–72 years; 21 males and 23 females). In Group I, 37 patients had a confirmed diagnosis of DVT (35 cases of lower limb thrombosis and two cases of upper limb thrombosis) and seven patients had a confirmed diagnosis of PE. The demographic and main clinical data of all patients are presented in Table 1. The results of performed laboratory tests are shown in Table 2.

**Table 1:** Comparison between the two studied groups according to demographic and clinical parameters.

	Total (*n* = 88)	Group II (*n* = 44)	Group I (*n* = 44)	Test of significance	*p*-value
Age (years) Mean ± SD.	58.50 ± 8.33	56.91 ± 9.17	60.09 ± 7.15	*t* = 1.816	0.073
Sex - n (%)					
Male	41 (46.6)	21 (47.7)	20 (45.5)	χ^2^ = 0.046	0.831
Female	47 (53.4)	23 (52.3)	24 (54.5)		
BMI (kg/m^2^) - Mean ± SD.	25.20 ± 5.90	24.55 ± 5.04	25.86 ± 6.65	*t* = 1.048	0.298
Smoking - n (%)	33 (37.5)	14 (31.8)	19 (43.2)	χ^2^ = 1.212	0.271
DM - n (%)	33 (37.5)	14 (31.8)	19 (43.2)	χ^2^ = 1.212	0.271
HTN - n (%)	31 (35.2)	15 (34.1)	16 (36.4)	χ^2^ = 0.050	0.823
Varicose veins - n (%)	8 (9.1)	3 (6.8)	5 (11.4)	χ^2^ = 0.550	^FE^*p*-value = 0.713
Oral contraceptive pills - n (%)	3 (3.4)	2 (4.5)	1 (2.3)	χ^2^ = 0.345	^FE^*p*-value = 1.000
Cancer stage - n (%)					
I	12 (13.6)	6 (13.6)	6 (13.6)	χ^2^ = 7.522	0.057
II	33 (37.5)	19 (43.2)	14 (31.8)		
III	30 (34.1)	17 (38.6)	13 (29.5)		
IV	13 (14.8)	2 (4.5)	11 (25.0)		
Cancer type - n (%)					
Breast	29 (33.0)	12 (27.3)	17 (38.6)	χ^2^ = 7.980	^MC^*p*-value = 0.416
Bladder	6 (6.8)	4 (9.1)	2 (4.5)		
Colon	25 (28.4)	15 (34.1)	10 (22.7)		
Gastric	14 (15.9)	8 (18.2)	6 (13.6)		
Lung	6 (6.8)	1 (2.3)	5 (11.4)		
Pancreatic	3 (3.4)	2 (4.5)	1 (2.3)		
Prostate	3 (3.4)	1 (2.3)	2 (4.5)		
Ovarian	1 (1.1)	1 (2.3)	0 (0.0)		
Brain	1 (1.1)	0 (0.0)	1 (2.3)		
Presence of thrombosis - n (%)	44 (50.0)	0 (0.0)	44 (100.0)	χ^2^ = 88.00^a^	<0.001^a^
VTE type - n (%)					
PE	–	–	7 (15.9)	–	–
DVT	–	–	37 (84.1)		
DVT site - n (%)					
Upper limb	–	–	2 (5.4)	–	–
Lower limb	–	–	35 (94.6)		
DVT Side - n (%)					
Unilateral	–	–	33 (89.2)	–	–
Bilateral	–	–	4 (10.8)		
Surgical tumor excision - n (%)	49 (55.7)	22 (50.0)	27 (61.4)	χ^2^ = 1.151	0.283
Central venous catheter - n (%)	41 (46.6)	12 (27.3)	29 (65.9)	χ^2^ = 13.198^a^	<0.001^a^
Systemic chemotherapy - n (%)	38 (43.2)	18 (40.9)	20 (45.5)	χ^2^ = 0.185	0.667

Qualitative data are described as numbers and percentages and compared using the chi-square test. Normally distributed quantitative data are expressed as means ± SD and compared using the student *t*-test.

BMI: Body mass index; DM: Diabetes mellites; HTN: Hypertension; VTE: venous thromboembolism; PE: Pulmonary embolism; DVT: Deep venous thrombosis; χ^2^: Chi square test; MC: Monte Carlo; FE: Fisher Exact; SD: Standard deviation; t: Student *t*-test; *p*-value comparing between non-VTE and VTE.

a Statistically significant at *p* ≤ 0.05.

**Table 2:** Comparison between the two studied groups according to laboratory parameters.

	Total (*n* = 88)	Group II (*n* = 44)	Group I (*n* = 44)	Test of Significance	*p*-value
Hemoglobin (g/dL) - Mean ± SD.	10.55 ± 0.71	10.51 ± 0.82	10.59 ± 0.57	*t* = 0.512	0.610
Platelets (× 10^3^/ μL) - Mean ± SD.	241.3 ± 65.99	235.5 ± 67.57	247.0 ± 64.64	*t* = 0.817	0.416
WBCs (× 10^3^/ μL) - Median (Min-Max)	5.75 (2.80–10.70)	6.0 (2.80–10.70)	5.55 (3.50–10.50)	*U* = 828.50	0.243
Urea (mg/dL) - Median (Min-Max)	86.0 (38.0–190.0)	87.0 (44.0–166.0)	83.0 (38.0–190.0)	*U* = 941.50	0.825
Creatinine (mg/dL) - Median (Min-Max)	1.85 (0.60–5.40)	1.65 (0.60–4.20)	2.15 (0.80–5.40)	*U* = 760.00	0.082
ALT (U/L) - Median (Min-Max)	66.0 (30.0–194.0)	65.50 (30.0–194.0)	71.50(36.0–194.0)	*U* = 846.00	0.308
AST (U/L) - Median (Min-Max)	66.0 (34.0–194.0)	61.50 (34.0–167.0)	71.50 (37.0–194.0)	*U* = 801.00	0.163
D-Dimer (mg/L) - Median (Min-Max)	1.91 (0.22–7.58)	0.87 (0.22–4.58)	3.01 (0.45–7.58)	*U* = 254.50^a^	<0.001^a^
ST2 (ng/mL) - Median (Min-Max)	9.92 (5.59–117.9)	8.56 (5.59–10.33)	13.02(7.65–117.9)	*U* = 51.00^a^	<0.001^a^

Normally distributed quantitative data are expressed as means ± SD and compared using the student *t*-test. Non-normally distributed data are expressed as medians (minimum-maximum) and compared using the Mann-Whitney test.

WBC: White blood count; ALT: alanine aminotransferase; AST: aspartate aminotransferase; ST2: soluble suppression of tumorigenicity 2; SD: Standard deviation; t: Student *t*-test; U: Mann-Whitney test.

*p*-value comparing Group I and Group II.

a Statistically significant at *p* ≤ 0.05.

Comparisons of the demographic data and clinical background between the two groups revealed that there was no statistically significant difference in age, sex, BMI, medical history or cancer stage between the two groups. Similarly, there was no significant difference between the two groups in the results of performed laboratory investigations including CBC parameters, urea, creatinine, ALT and AST.

**Figure 1:**
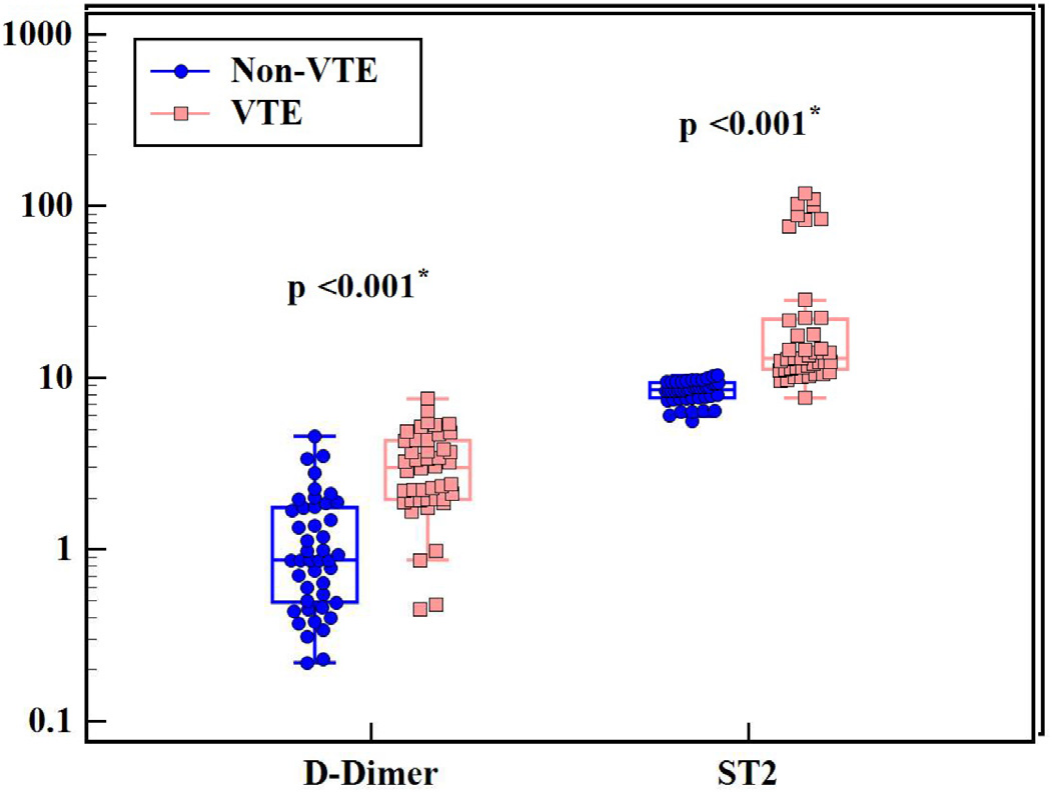
Comparison between the two studied groups according to D-Dimer and soluble suppression of tumorigenicity 2 using the Mann-Whitney test (data are presented as medians, Interquartile range, minimum, maximum and outliers).

Regarding D-dimer results, values were significantly higher in VTE patients compared to non-VTE patients (*p*-value < 0.001 - Figure 1 and Table 2). Median D-dimer was 3.01 mg/L (range: 0.45–7.58 mg/L) in Group I and 0.87 mg/L (range: 0.22–4.58 mg/L) in Group II. D-dimer was positively correlated with cancer stage (*r* = 0.722; *p*-value < 0.001) with significantly higher D-dimer levels being associated with advanced cancer stages. D-dimer values were also correlated with cancer type (*H* = 14.318; *p*-value = 0.026) with the highest levels being observed in lung and pancreatic cancer cases.

**Figure 2:**
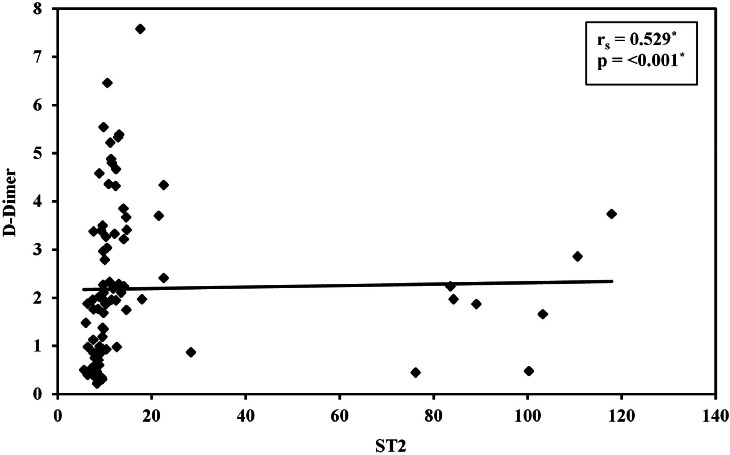
Correlation between soluble suppression of tumorigenicity 2 and D-Dimer in all subjects. Spearman coefficient = 0.529; *p*-value < 0.001.

Median serum sST2 was 13.02 ng/mL (range: 7.65–117.9 ng/mL) in Group I and 8.56 ng/mL (5.59–10.33 ng/mL) in Group II. Serum ST2 levels were significantly higher in VTE compared to non-VTE patients (*p*-value < 0.001 - Figure 1 and Table 2). A positive correlation was found between Serum ST2 and D-dimer levels (*r* = 0.529; *p*-value < 0.001 - Figure 2). The correlations of serum ST2 with cancer stage and cancer type were not statistically significant. When examining thrombotic risk factors (other than malignancies) in all study subjects, both D-dimer and sST2 had significant positive correlations with the presence of central venous catheter (*U* = 182.00 with *p*-value < 0.001 and *U* = 621.00 with *p*-value = 0.004, respectively) and D-dimer was significantly higher (*p*-value = 0.011) in smokers (Table 3).

**Table 3:** Relationship between sST2 and D-dimer with different thrombosis-related parameters in all subjects.

	n	ST2 (ng/mL)	Test of Sig (*p*-value)	D-Dimer (mg/L)	Test of Sig (*p*-value)
		Median (range)		Median (range)	
DM					
Negative	55	10.03 (6.0–117.9)	*U* = 840.0 (0.561)	1.76 (0.22–7.58)	*U* = 781.0 (0.276)
Positive	33	9.76 (5.59–89.08)		1.97 (0.23–6.46)	
HTN					
Negative	57	9.78 (6.32–117.9)	*U* = 816.0 (0.555)	1.75 (0.22–5.54)	*U* = 711.0 (0.132)
Positive	31	10.07 (5.59–89.08)		2.02 (0.40–7.58)	
Smoking					
Non-smoker	55	9.55 (5.59–110.6)	*U* = 708.50 (0.086)	1.48 (0.22–6.46)	*U* = 612.5^a^ (0.011^a^)
Smoker	33	11.03 (6.44–117.9)		2.24 (0.49–7.58)	
Varicose veins					
Negative	80	9.79 (6.0–117.9)	*U* = 296.50 (0.733)	1.91 (0.23–7.58)	*U* = 274.50 (0.509)
Positive	8	12.01 (5.59–83.57)		2.06 (0.22–3.41)	
Cancer stage					
I	12	11.02 (6.32–100.3)	*H* = 5.246 (0.155)	0.47 (0.22–2.11)	*H* = 47.369^a^ (<0.001^a^)
II	33	9.36 (5.59–103.2)		0.99 (0.34–3.70)	
III	30	9.76 (6.00–117.9)		2.07 (0.55–4.67)	
IV	13	11.51 (8.83–110.6)		4.88 (2.86–7.58)	
r_s_ (*p*-value)		0.112 (0.298)		0.722^a^ (<0.001^a^)	
Cancer type					
Breast	29	10.51 (5.59–110.6)	*H* = 5.719 (0.455)	0.98 (0.23–5.22)	*H* = 14.318^a^ (0.026^a^)
Bladder	6	7.80 (6.0–14.70)		1.34 (0.44–3.41)	
Colon	25	9.68 (6.34–117.9)		1.88 (0.22–7.58)	
Gastric	14	9.76 (7.56–17.92)		1.87 (0.49–4.32)	
Lung	6	10.96 (10.03–14.56)		4.28 (1.93–6.46)	
Pancreatic	3	9.56 (8.83–11.51)		4.58 (1.38–4.80)	
Prostate	3	11.03 (8.82–14.11)		2.24 (2.02–2.33)	
Ovarian	1	9.36^#^		0.34^#^	
Brain	1	22.54^#^		2.41^#^	
Surgical tumor excision					
No	39	9.76 (5.59–110.62)	*U* = 886.50 (0.562)	1.75 (0.22–7.58)	*U* = 812.50 (0.230)
Yes	49	10.25 (6.00–117.88)		1.97 (0.37–5.33)	
Central venous catheter					
Absent	47	8.90 (5.59–100.29)	*U* = 621.00^a^ (0.004^a^)	0.87 (0.22–3.70)	*U* = 182.00^a^ (<0.001^a^)
Present	41	11.19 (6.00–117.88)		3.33 (0.45–7.58)	
Systemic chemotherapy					
Absent	50	9.65 (6.32–110.62)	*U* = 938.00 (0.919)	1.72 (0.22–7.58)	*U* = 806.00 (0.225)
Present	38	10.19 (5.59–117.88)		1.97 (0.46–6.46)	

Sig: Significance; DM: Diabetes mellitus; HTN: Hypertension; SD: Standard deviation; H: H for Kruskal-Wallis test; U: Mann-Whitney test; r_s_: Spearman coefficient.

*p*-value for relationship between ST2 and different parameters, relation between D-dimer and different parameters.

a Statistically significant at *p*-value ≤0.05.

**Figure 3:**
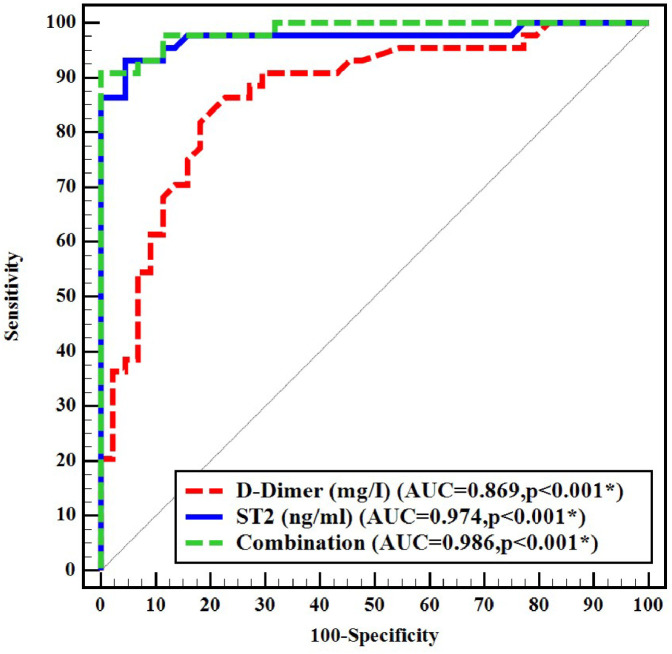
Receiver operating characteristic (ROC) curve for D-Dimer and soluble suppression of tumorigenicity 2 to discriminate venous thromboembolism from non-venous thromboembolism cases.

ROC curve analysis was performed to evaluate the diagnostic performance of D-dimer and sST2. D-dimer exhibited an area under the curve (AUC) of 0.869. Using the manufacturer cutoff value of 0.5 mg/L for D-dimer, the sensitivity was 95.5 % and the specificity was 27.3 %. The best D-dimer cutoff value obtained from the ROC curve was 2.02 mg/L which showed 70.45 % sensitivity and 86.36 % specificity. The best cutoff point for sST2 was 10.033 ng/mL, with an AUC of 0.974, sensitivity of 93.18 %, and specificity of 95.45 %. The combined use of D-dimer and sST2 yielded an AUC of 0.986, with sensitivity and specificity of 90.9 % and 93.2 % respectively (Figure 3 and Table 4).

**Table 4:** Diagnostic performance for D-dimer and ST2 to identify venous thrombosis.

	AUC	*p*-value	95 % CI	Cut off	Sensitivity	Specificity	PPV	NPV
D-dimer	0.869	<0.001^a^	0.792–0.945	>0.5	95.5	27.3	56.8	85.7
				>2.02^b^	70.45	86.36	83.8	74.5
ST2	0.974	<0.001^a^	0.938–1.000	>10.033	93.18	95.45	95.3	93.3
D-dimer + ST2	0.986	<0.001^a^	0.968–1.000		90.9	93.2	93.0	91.1

AUC: Area Under a Curve; 95 % CI: 95 % Confidence interval; NPV: Negative predictive value; PPV: Positive predictive value; ST2: soluble suppression of tumorigenicity 2.

a Statistically significant at *p*-value ≤ 0.05.

b Cut off was chosen according to the Youden index.

## Discussion

Identification of new biomarkers for the diagnosis of VTE in cancer patients is an important research focus.^1^^,^^2^ Despite its high sensitivity and high negative predictive value (NPV), the usefulness of D-dimer test in cancer patients is limited by its low specificity and its high baseline values in malignancies.^17^

Apart from its role in inflammatory conditions, sST2 has been evaluated in many other disorders including acute and chronic heart failure, myocardial infarction, atrial fibrillation, bronchial asthma and stroke.^12^^,^ 18, 19, 20 Higher concentrations of sST2 were associated with disease progression and predicted poor prognosis in these disorders.^12^^,^^18^^,^^19^ The interleukin 33-ST2 system has recently been shown to induce expression and activity of tissue factor.^21^ This provides an explanation for the strong association of sST2 with ischemic stroke, myocardial infarction and systemic embolism in atrial fibrillation patients.^14^^,^^15^^,^^20^ To the best of our knowledge, sST2 has been studied only once as a marker of VTE.^9^

The present work focused on the association between the sST2 level and hypercoagulable state in cancer-associated VTE. Both D-dimer and sST2 were significantly higher in patients with VTE than in Group II. However, sST2 showed the advantage of higher specificity and positive predictive value (PPV).

Regarding D-dimer, the results of this study were comparable with previous research that studied D-dimer in CAT with significantly higher test values in VTE cancer patients. The conventional cutoff value showed poor specificity and low PPV. Among these studies, Qdaisat et al. showed that D-dimer levels were significantly higher in VTE than in non-VTE cancer patients. Although D-dimer sensitivity was high, the specificity was low both using the conventional cutoff and 75th percentile cutoff point of the study population. In addition, the D-dimer diagnostic accuracy was variable in different tumor types.^17^ Koch et al. also reported D-dimer values to be significantly higher in VTE than non-VTE cancer patients. Their study also confirmed that the use of 0.5 mg/L cutoff had a good sensitivity with low specificity (65 %). This could be improved by raising the cutoff point to 4.9 mg/L (95 % specificity and 64 % sensitivity).^22^ Our results showed that D-dimer levels were positively correlated with cancer stage which affects the diagnostic usefulness of this test. This finding was similar to previous studies evaluating D-dimer in different types of cancer. Siddiqui Dai et al. found a positive correlation between increased D-dimer values and advanced cancer stage in different cancer types.^23^ Lee et al. reported a positive association between D-dimer and the TNM stage in patients with colorectal cancer^24^ and the study by Dirix et al. showed similar results in breast cancer patients.^25^ Furthermore, Mego et al. showed a strong association between the D-dimer test, lymphovascular invasion and the presence of circulating tumor cells in breast cancer patients.^26^ The results of the present study showed that D-dimer levels were strongly associated with cancer type, with the highest values observed in lung cancer and pancreatic cancer patients; both types of cancer are considered high-risk cancer types for developing VTE.^27^

Although the associations between sST2 and both cancer type and cancer stage were statistically non-significant, this is the first study to investigate these associations and so this promising finding requires confirmation through further studies recruiting a larger number of cancer patients with different types and stages of malignancy. Soluble ST2 in VTE was first investigated by Memon et al. who studied the plasma levels of a panel of protein biomarkers in DVT and non-DVT patients. Seven proteins, including the sST2 protein, were significantly higher in patients with DVT. The study showed a strong correlation between D-dimer and the plasma levels of these biomarkers.^9^ sST2 was strongly correlated with other studied biomarkers including: activated protein C-protein-C inhibitor complex (APC-PCI), osteopontin, P-selectin, tissue factor pathway inhibitor (TFPI), transferrin receptor protein-1 and von Willebrand factor (vWF). However, the study did not investigate the association between these biomarkers and different risk factors of thrombosis.^9^ To the best of our knowledge, the present work is the first to investigate the correlation between the serum sST2 level and thrombotic risk factors encountered in the studied cancer patients. The only statistically significant association detected was with the presence of a central venous catheter. Negligible and non-significant associations were detected with diabetes mellitus, hypertension, BMI, varicose veins, smoking, cancer type, cancer stage and systemic chemotherapy.

## Study limitations

The main limitation in this study was the small sample size, the low number of PE cases and the lack of cases with both DVT and PE. Another limitation was the absence of other types of cancer, such as bone, uterine and testicular cancer. Future multicenter studies, with larger numbers of patients and a broader range of cancer types, are required to confirm these results. Another limitation was the unavailability of test results for other hemostasis parameters for all the patients, so we could not include other hemostasis parameters in the results.

## Conclusion

To the best of our knowledge, the present study is the second study that supports the potential role of sST2 in VTE, and the first to study sST2 in CAT. The levels of this novel serum biomarker were positively correlated with D-dimer levels in patients, however, with greater specificity and higher PPV. Further research is encouraged to confirm the results regarding sST2 in CAT. In addition, further research is recommended enrolling larger numbers of cancer patients with VTE to determine the best cutoff values for sST2 to diagnose thrombosis in different types of cancer.

## Funding

None to declare.

## Authors’ contributions

EMA developed the study design and wrote the first draft of the manuscript. EMA, AMD and EHM acquired the research data, contributed to the practical part, and made critical revisions to the paper. All authors finally approved the paper for submission.

## Conflicts of interest

None to declare.
